# Peri-Ictal Changes in Depression and Anxiety in Persons With Epileptic and Non-epileptic Seizures

**DOI:** 10.3389/fpsyt.2022.912697

**Published:** 2022-07-22

**Authors:** Jennifer Hopp, Autusa Pahlavan, Mary Richert, Kathryn Grimes, Kate Turlington, Maureen Cassady, Mark D. Kvarta, Scott M. Thompson

**Affiliations:** ^1^Division of Epilepsy, Department of Neurology, University of Maryland School of Medicine, Baltimore, MD, United States; ^2^Department of Pediatrics, University of Virginia, Charlottesville, VA, United States; ^3^Department of Psychiatry, University of Maryland School of Medicine, Baltimore, MD, United States; ^4^Department of Physiology, University of Maryland School of Medicine, Baltimore, MD, United States

**Keywords:** generalized epilepsies, psychogenic seizure, depression, anxiety, focal epilepsies

## Abstract

**Objective:**

We tested the hypothesis that epileptic, but not non-epileptic, seizures would produce an improvement in comorbid depression and anxiety symptoms in the peri-ictal period, much like the antidepressant effects of electroconvulsive therapy.

**Methods:**

We examined depression and anxiety symptoms in patients admitted to an inpatient unit for continuous video electroencephalography as part of routine clinical care. Patients completed three questionnaires that included the Beck Depression Inventory-II (BDI), Montgomery Asberg Depression Rating Scale (MADRS), and Beck Anxiety Inventory (BAI) after admission, in the 24 h following a seizure, then again 2 weeks after the last seizure.

**Results:**

In patients with epilepsy, depression and anxiety scores improved in the 24 hrs following a seizure (change in BDI = 24%; change in MADRS = 19%; change in BAI = 21%) but returned toward baseline after 2 weeks. In patients with non-epileptic seizures, depression and anxiety scores also improved in the 24 hrs following a psychogenic non-epileptic seizure (change in BDI = 17%, change in MADRS = 27%, change in BAI = 36%). There was a greater improvement in depression and anxiety scores in patients with focal-onset epilepsy (BDI = 30%, MADRS = 22%, BAI = 30%) compared to generalized seizure onset (BDI = 6%, MADRS = 12%, BAI = 8%).

**Conclusion:**

We conclude that single seizures can result in transient improvements in mood. Because seizures need not be generalized or epileptic to exert this effect, the underlying mechanisms are uncertain.

## Introduction

Depression and anxiety are common comorbidities in epilepsy, affecting 40–60% of patients with epilepsy (1–5). Depressive symptoms are correlated with seizure type, seizure frequency, suboptimal pharmacological treatment, and lack of social activity (6). Depression is underdiagnosed (4, 7) and can be inadequately managed, resulting in increased cost and poor quality of life (8, 9). Furthermore, suicide rates in persons with epilepsy are five to ten times higher than the general population (7). Although the comorbidity of mood disorders and epilepsy is well-recognized, peri-ictal changes in psychiatric symptoms are less well characterized and measured (10, 11).

Electroconvulsive therapy (ECT) is an effective treatment for severe depression (12). The antidepressant efficacy of ECT is thought to depend on induction of generalized seizures (13) and is positively correlated with seizure spread. By analogy to ECT, we hypothesized that patients with epilepsy and depression would experience an improvement in mood following a single seizure and that patients with generalized epilepsy would experience a greater mood improvement after a seizure than patients with focal epilepsy due to greater spread of seizure activity.

Patients with psychogenic non-epileptic seizures (PNES) also have comorbid depression and anxiety (7, 14–18). PNES patients with more psychiatric comorbidity face poorer outcomes (19). If the effects of epileptic seizures on mood are due to ECT-like changes in neuronal activity, we predicted that PNES patients would not experience improved mood immediately after seizures because they do not experience abnormal epileptiform neuronal discharge.

We report here the transient improvement of depression scores in the 24 h following focal epileptic seizures, and transient improvement in depression and anxiety in patients after psychogenic non-epileptic seizures.

## Materials and Methods

We enrolled patients admitted to an inpatient epilepsy unit for video-EEG monitoring as part of their routine clinical care. We administered standard self-reporting mood questionnaires before and after defined seizure events and later defined and classified those events as epileptic and non-epileptic.

### Study Design and Participants

Subjects in this study were recruited from adult patients of 18 years of age or older who were admitted to the Epilepsy Monitoring Unit (EMU) at the University of Maryland Medical Center as part of routine clinical care between June 2014 and July 2018. All subjects gave their consent to participate. Subjects had to score ≥ 24 on the mini mental status examination and meet cognitive and reading criteria to be able to understand and complete the psychological questionnaires. If they could not read, this was offered to them. Antiseizure medications were discontinued in the EMU (Table 1), but not antidepressants. All procedures were approved by the University of Maryland Institutional Review Board. Of 696 patients admitted to the EMU during this time period, 181 eligible patients were approached about participating, 156 consented, and 139 were enrolled. The 17 patients who met inclusion criteria and consented but did not enroll did not do so either because they changed their mind after consent or were discharged before enrollment could occur. Of the 139 enrolled during this time period, 64 had a seizure during their inpatient stay in the EMU and completed at least one mood assessment.

**TABLE 1:** Antiseizure medications discontinued.

Medication	# Patients
Levetiracetam	14
Carbamazepine	6
Lacosamide	5
Lamotrigine	4
Phenytoin	4
Topiramate	3
Benzodiazepine	3
Zonisamide	2
Pregabalin	1
Eslicarbazepine	1
Oxcarbazepine	1
Phenobarbital	1

*The graph provides the name of the medication discontinued and the number of patients who discontinued that medication.*

### Mood and Anxiety Assessments

Patients’ mood was assessed using the Beck Depression Inventory-II (BDI), Beck Anxiety Inventory (BAI), and the Montgomery-Asberg Depression Rating Scale (MADRS). Patients were not interviewed by a psychiatrist. Because the MADRS takes longer to administer than the BDI, some patients failed to complete the MADRS and were only administered the BDI. The BDI is known to yield results with consistent, high re-test reliability (20). Incomplete questionnaires were not scored. Ranges for the descriptive labels were: 0–13 = minimal, 14–19 = mild, 20–28 = moderate, and 29–63 = severe for the BDI, 0–7 = minimal, 8–15 = mild, 16–25 = moderate, and 26–63 = severe for the BAI, and 1–6 = normal, 7–19 = mild, 20–34 = moderate, and >34 = severe for the MADRS (21, 22). Baseline scores were obtained within 24 hrs of admission to the EMU and before the occurrence of a seizure. Only patients with scores above the cutoff for minimal depression or anxiety were included in examining the effects of seizures on mood and anxiety, although they were included in the patient demographics. The BDI, BAI, and MADRS were administered 4 h, 12 h, 24 h, and 2 weeks following their last seizure event, as determined from the video-EEG monitoring. Only a minority of participants (26 and 23 for BDI and BAI, respectively) were able to complete all the first three post-seizure questionnaires due to patient and logistical factors. As a result, we computed an average of all scores obtained during this window and refer to this value as “24-h post-seizure” in all analyses. To validate that this did not bias our result, we determined the test-retest correlation across these timepoints for both anxiety and depression measures across the study sample. The test-retest *r* was 0.68–0.90 for BAI between each of these timepoints (*p* < 5 × 10^–5^) and *r* = 0.76–0.85 for BDI (*p* < 2 × 10^–6^) indicating these measures were in very good agreement with each other during this 24 hr time period (23). The 2-week follow-up questionnaires were administered *via* a phone interview, although we succeeded in contacting only a subset of patients. The MADRS was not assessed for all patients and not during the 2-week phone call due to difficulties in administering it over the phone.

### Seizure Diagnosis

Patients underwent continuous video EEG monitoring and clinical assessment by a board-certified epileptologist during their stay as part of routine clinical care. The epileptologist classified the seizures as epileptic (ES) or non-epileptic (NES). They also determined whether the NES were psychogenic or physiologic and classified ES as focal or generalized, as well as localized focal onset seizures if possible. Physiologic NES patients, whose seizure diagnosis was attributed to side effects of medications or other organic causes, were excluded from all analyses (*n* = 6).

Statistical analyses were performed using paired and unpaired *t*-tests, one-way repeated measures ANOVA, and linear regression with SigmaStat software.

## Results

### Incidence of Mood and Anxiety Symptoms in Patients With Seizures

Of the 139 patients enrolled in the study, 33 patients (24%) had epileptic seizures (ES) and 31 patients (22%) had non-epileptic seizures (NES). Of the NES patients, six were physiologic and the remaining 25 had psychogenic NES (PNES). The remaining 75 patients (54%) did not have a seizure during their time in the EMU or failed to complete any baseline questionnaires. The six physiological PNES patients were not included in subsequent analyses. Of the patients with epileptic seizures, 25 patients had focal-onset seizures (16 temporal, 5 frontal, 2 central, 2 of unknown onset), and 8 patients had generalized seizures. Secondarily generalized seizures occurred in 8 of the patients with focal epilepsy. Table 2 shows demographic information of patients with epileptic and non-epileptic seizures, with a majority of Caucasian females with an average age in the mid-to-late 30 s.

**TABLE 2:** Baseline patient demographic information.

	Epileptic	Non-epileptic
Number enrolled	33	31
Age (years)	39.1 ± 2.5	35.0 ± 2.3
**Sex**		
Female	23 (70%)	26 (84%)
Male	10	5
**Ethnicity**		
Caucasian	18 (55%)	19 (61%)
Black/African-American	12	10
Asian	1	0
Other	2	2
Number of seizure events	2.9 ± 0.6	2.0 ± 0.4
**Past psychiatric diagnoses**		
Anxiety	9 (27%)	13 (42%)
Depression	14 (42%)	13 (42%)
Bipolar disorder	1	0
Posttraumatic stress disorder	0	4
Obsessive compulsive disorder	0	2
Attention deficit disorder	1	0

*Data are number or mean ± SEM for eligible patients who had a seizure in the EMU. Ethnicity and past psychiatric history taken from patient self-reporting.*

**FIGURE 1:**
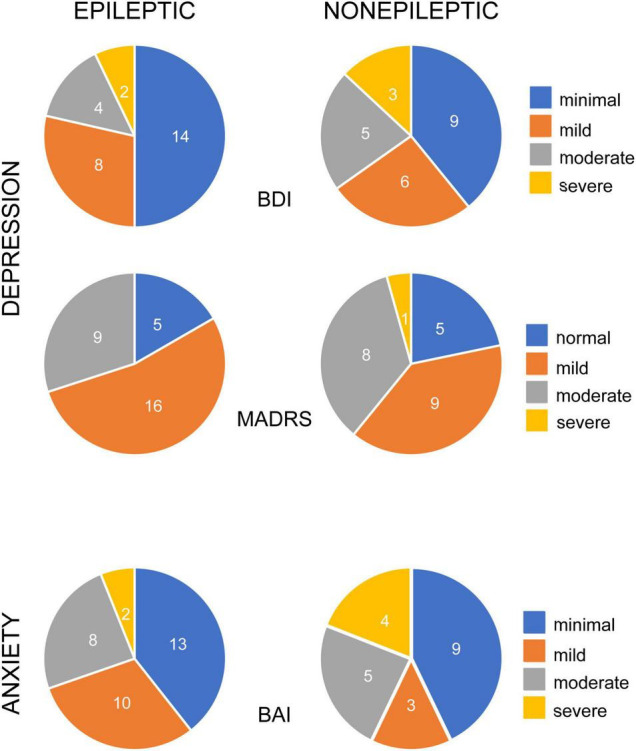
Occurrence of depression and anxiety symptoms in patients with epileptic and non-epileptic seizures. Pie charts illustrate the percentage of epileptic (left column) and non-epileptic (right column) patients with self-reported symptoms, color coded for degree of severity, as indicated. Depression symptoms were evaluated with the Becks Depression Index-II (BDI, upper row, *n* = 28 ES, 23 NES) and the Montgomery-Asberg Depression Rating Scale (MADRS, middle row, *n* = 30 ES, 23 NES). Anxiety symptoms were evaluated with the Beck Anxiety Inventories (BAI, bottom row, *n* = 33 ES, 21 NES).

Many patients with epileptic and non-epileptic seizures showed evidence of elevated depression and anxiety scores on all indices at baseline (Figure 1). Depression was severe or moderate in 21% (BDI) or 30% (MADRS) of the patients with ES and 34% (BDI) and 39% (MADRS) of the patients with NES. Among patients with ES, there was no significant difference in mean baseline BDI or MADRS score between those with focal seizures lacking secondarily generalized activity and generalized seizures (BDI: 15.8 ± 2.4 vs. 12.6 ± 2.4, *n* = 17,9; *p* = 0.3598; MADRS: 16.1 ± 2.3 vs. 12.8 ± 2.4, *n* = 15, 9; *p* = 0.3353; unpaired *t*-tests).

Baseline anxiety scores were severe or moderate in 30% of the ES patients and 43% of the NES patients. There was no significant difference in baseline anxiety scores between ES and NES patients (BAI: 10.7 ± 1.5 vs. 15.0 ± 2.7; *n* = 35, 23; *p* = 0.13; unpaired *t*-test). There was no significant difference in BAI scores between ES patients with focal seizures, without secondarily generalized seizures, and generalized seizures (*p* = 0.7970).

**FIGURE 2:**
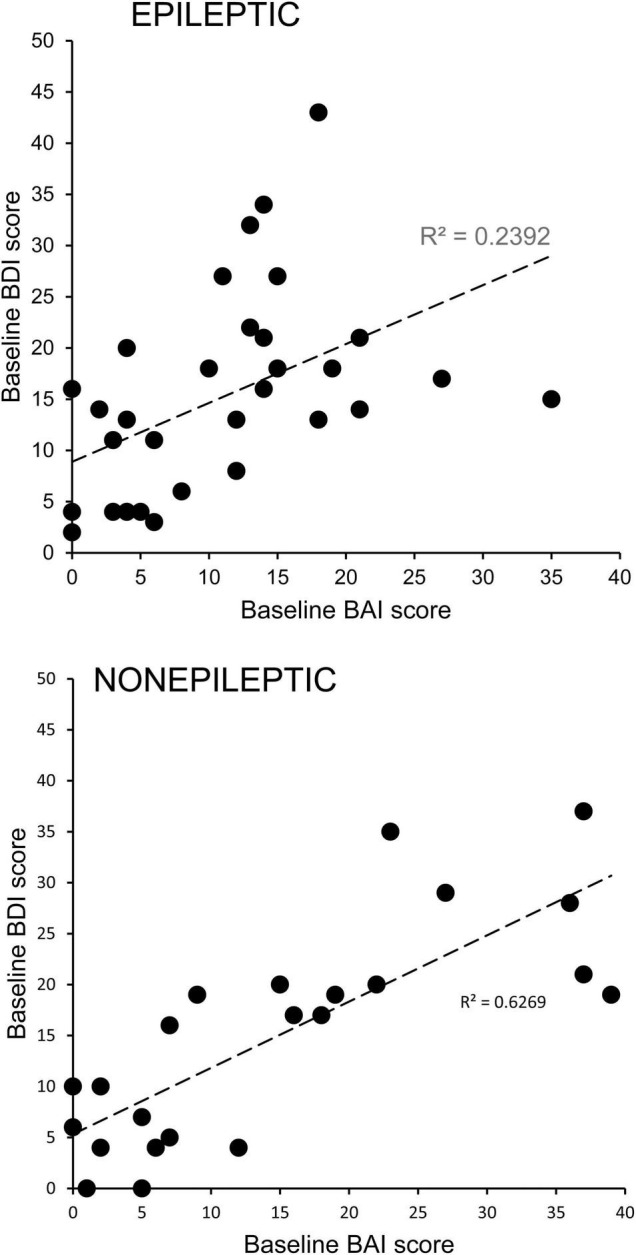
Comorbidity of depression and anxiety symptoms in patients with epileptic and non-epileptic seizures. Depression scores for individual patients with epileptic (upper graph) or non-epileptic (lower graph) seizures as assessed with the Becks Depression Index-II (BDI) are plotted as a function of their anxiety scores as assessed with the Beck Anxiety Inventories (BAI). The positive correlations were significant for both groups (ES, Pearson *p* = 0.0061; NES, Pearson *p* < 0.00001), indicating a high degree of comorbidity.

There were significant positive correlations between the BDI and BAI scores of individual ES (*R*^2^ = 0.2392, Pearson *p* = 0.0061) and NES (*R*^2^ = 0.6269, Pearson *p* < 0.00001) patients (Figure 2), highlighting the comorbidity of these symptoms.

### Effects of Seizures on Mood and Anxiety

**FIGURE 3:**
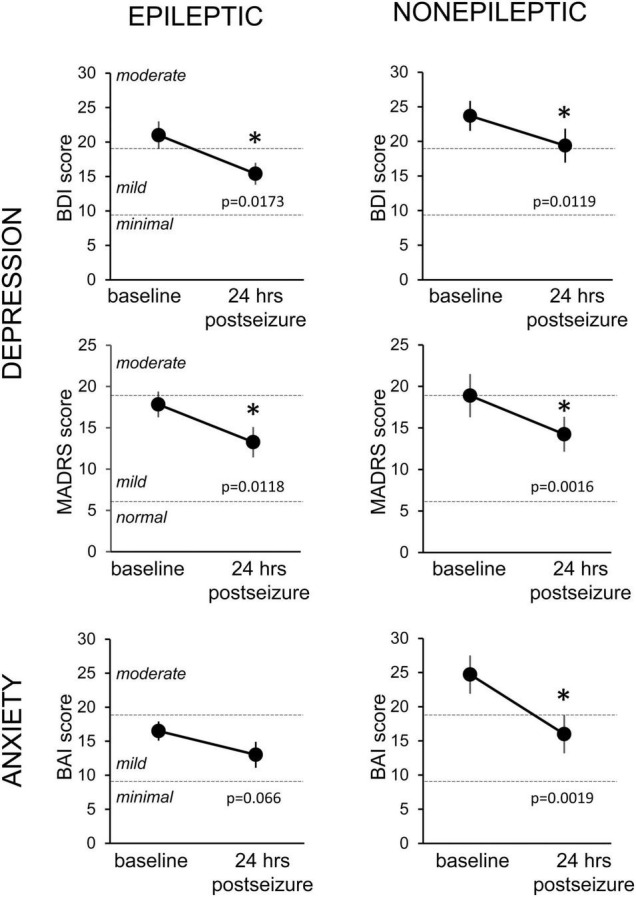
Changes in depression and anxiety symptoms in the 24-h period following an epileptic or non-epileptic seizure. Depression symptoms as assessed with the Becks Depression Index-II (BDI, upper row, *n* = 15 ES, 14 NES) and the Montgomery-Asberg Depression Rating Scale (MADRS, middle row, *n* = 25 ES, 18 NES) at baseline, upon entry to the EMU, and in the 24 h following an epileptic (left column) or non-epileptic (right column) seizure. Anxiety symptoms were evaluated with the Beck Anxiety Inventories (BAI, bottom row, *n* = 20 ES, 14 NES). Statistically significant improvements in mood scores were seen in all groups except the BAI following epileptic seizures. Data presented as mean ± SEM. * Statistically significant decrease.

To determine the effect of seizures on depression and anxiety scores, we excluded patients who had mood scores indicating no depressive symptoms or minimal symptoms at baseline testing from the analysis and included only patients with at least mild severity scores. We found that mood scores decreased during this 24-h period for both ES and NES patients across all three measures (Figure 3). This improvement in mood and anxiety was statistically significant using paired *t*-tests for ES patients on the BDI and MADRS and for NES patients on the BDI, MADRS and BAI. The average decrease from the baseline score was 23.6 ± 8.5% in the BDI, 18.8 ± 13.0% in the MADRS, and 20.6 ± 11.3% in the BAI for ES patients (*n* = 15, 25, 20) and for NES patients it was 16.7 ± 7.1% in the BDI, 26.8 ± 6.6% in the MADRS, and 35.8 ± 6.5% in the BAI (*n* = 15, 18, 14).

It is possible that discontinuation of antiseizure medications upon entry to the EMU as part of standard care could have an independent effect on mood. This is difficult to quantify due to the array of AED medication types (Table 1) and the observational nature of the study. The most commonly tapered medication in our study was levetiracetam, a medication which can contribute to mood destabilization (24). We tested whether there was a difference in mood or anxiety scores at the first timepoint after seizure compared to baseline in those who had levetiracetam tapered (*n* = 27/61) vs. those who did not. There was no significant difference in the total patient sample for anxiety (*t*_(46)_ = 0.025, *p* = 0.98) or depression (*t*_(46)_ = 0.38, *p* = 0.70) scores. This did not differ when stratified by seizure type (*p* = 0.51–0.98).

To further characterize the effect of seizures on mood, we analyzed the differences in mood scores based on different seizure types in patients with ES. Patients with focal-onset seizures without secondarily generalized activity, had a significant decrease in mood scores on the BDI (44%, *p* = 0.0133) and MADRS (41%, *p* = 0.0008), but a not significant decrease on the BAI (31%, *p* = 0.4375) measure. In contrast, patients with generalized-onset seizures did not have a significant change in mood scores on any of the three questionnaires, although the sample size is small (*n* = 4–7).

**FIGURE 4:**
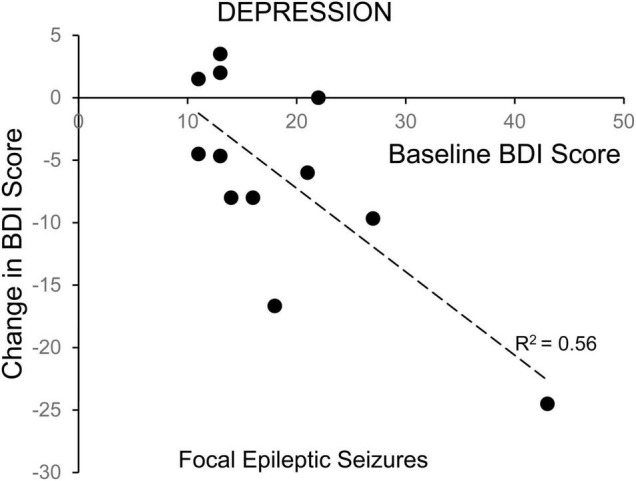
Correlation of change in depression symptoms with baseline symptom severity for patients with focal epileptic seizures. The change in depression symptoms for individual patients, as evaluated with the Becks Depression Index-II, in the 24-h following a focal epileptic seizure, is plotted as a function of their baseline symptom severity. The correlation was statistically significant (Pearson *p* = 0.0048).

Linear regression revealed a significant correlation between starting score and 24-h change in BDI depression scores for ES patients with focal seizures without secondarily generalized activity (Figure 4) (*R*^2^ = 0.56, *p* = 0.0048), but not for ES patients with generalized seizures or NES patients. There was also no statistical correlation between starting BAI scores and 24-h change for the ES or NES patients. Patients with more severe depression at baseline experienced a larger improvement in mood scores in the 24 h following a focal seizure. Because our hypothesis focused on epilepsy with comorbid mood symptoms, we excluded those with a low-symptom burden in the primary analysis. However, we performed secondary analysis on this group to test the possibility that symptoms could increase as well, especially in those with few symptoms at baseline. There was no significant change in BDI, MADRS, or BAI in individuals with NES (*p* > 0.7 for all). Similarly, there was no change in BDI or MADRS scores for individuals with ES, except for a modest increase in reported BAI scores in this group of individuals with a low baseline symptom burden (2.9 ± 2.1 to 4.9 ± 3.5, *p* = 0.031).

**FIGURE 5:**
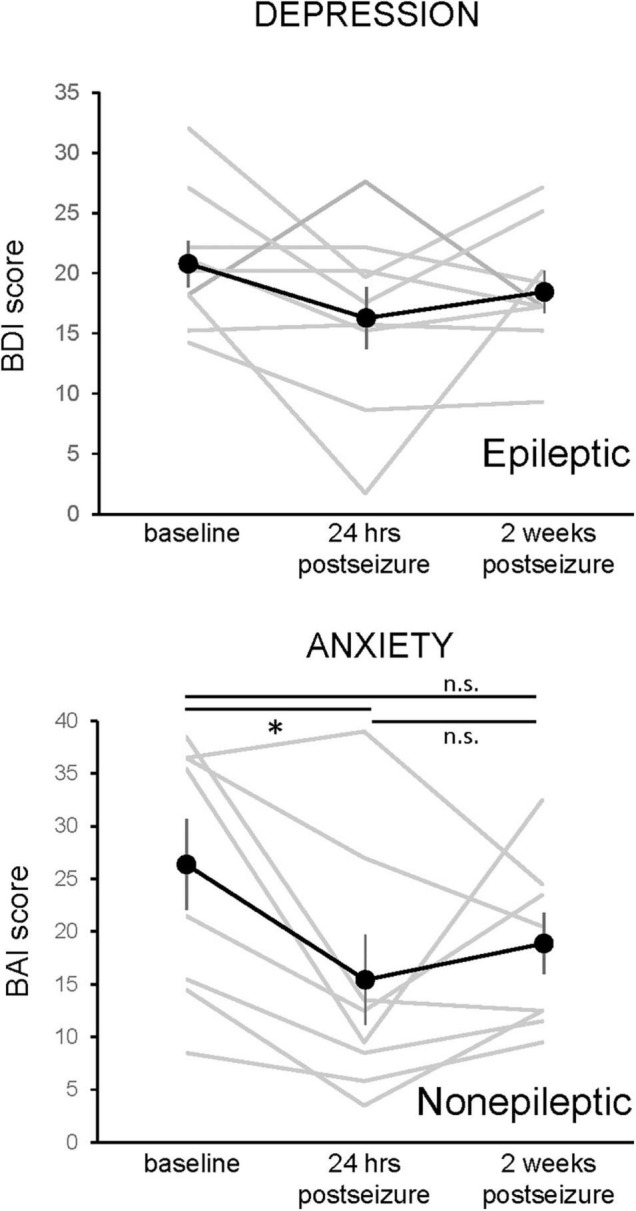
Changes in depression and anxiety symptoms 24-h and 2 weeks following an epileptic or non-epileptic seizure. Depression symptoms, as assessed with the Becks Depression Index-II (BDI, upper graph, *n* = 9), and anxiety symptoms, as assessed with the Beck Anxiety Inventories (BAI, lower graph, *n* = 8), are shown at baseline, in the 24 h following an epileptic (BDI) or non-epileptic (BAI) seizure, and 2 weeks after the last seizure. The effects of epileptic seizure on the BDI score were not statistically significant (rmANOVA). There was a main effect of non-epileptic seizures in the BAI (rmANOVA, *F*_(1.97,13.42)_ = 4.657, *p* = 0.0303). Paired *post-hoc* comparisons revealed that the 24-h postseizure time point was significantly lower than the baseline value and that the 2-week time point was not different from either baseline or 24 h postseizure. Data presented as mean ± SEM, with responses of individual patients shown in gray.

After noting an improvement in mood scores in the 24 h after seizure activity, we then assessed whether this improvement persisted 2 weeks after a seizure. Unfortunately, only a minority of patients responded to the request to complete the questionnaires by telephone (9 of 13 ES patients, 8 of 14 NES patients). Figure 5 shows the 2-week change in mood scores for the subset of ES patients on the BDI and NES patients on the BAI. A one-way repeated measures ANOVA showed a main effect for the BAI results in NES patients (*F*_(1.917,13.42)_ = 4.657, *p* = 0.0303, *n* = 8), with a significant decrease at the 24-h time point. The 2-week time point was not significantly different from either the baseline or 24-h time point. There was no main effect for the BDI in patients with ES (*F* = 1.985, *p* = 0.170, *n* = 9).

**FIGURE 6:**
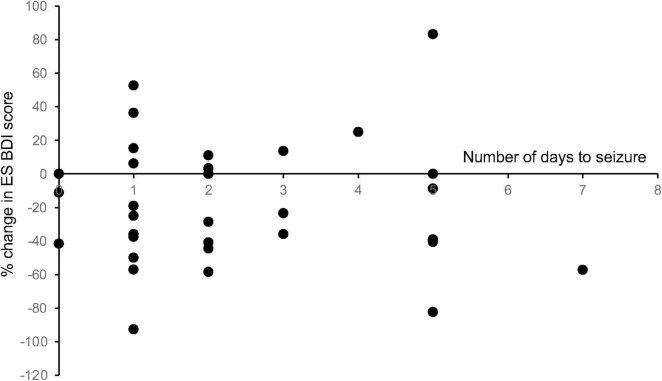
Length of stay in the EMU compared with change in mood scores. To determine whether the length of stay in the EMU had any effect on the change in mood scores, we performed a linear regression analysis of the number of days admitted to the EMU prior to the first seizure event versus the change in mood score. This correlation was found to be non-significant across all mood measures for patients with both ES and NES (R2 values: ES BDI = 0.0002, ES BAI = 0.025, ES MADRS = 0.083, NES BDI = 0.030, NES BAI = 0.0029, NES MADRS = 0.014).

To determine whether the length of stay in the EMU had any effect on the change in mood scores, we performed a linear regression analysis of the number of days admitted to the EMU prior to the first seizure event versus the change in mood score. This correlation was found to be non-significant across all mood measures for patients with both ES and NES (Figure 6).

## Discussion

### Comorbidity of Seizures and Mood Disorders

The relationship between depression and epilepsy, as well as specifically between seizures and mood, is likely complex and multifactorial. Persons with epilepsy have a higher risk of developing depression and patients with depression have a higher risk for epilepsy and poorer outcomes (4). The incidence of depression and anxiety is reported to be about twice as high in patients with epilepsy compared to the general population (25). Similarly, anxiety disorders are known to affect 10–25% of persons with epilepsy, compared to less than 6% in the general population (2). We observed an incidence of moderate-to-severe depression and/or anxiety scores in persons with epilepsy of 20–40% in our patient population, consistent with these previous studies. Furthermore, in individual ES and NES patients we found a high correlation between baseline depression and anxiety scores, highlighting the strength of the comorbidity. The incidence of depression has been reported previously to be greater in patients with temporal lobe and other focal epilepsies (54–60%) than among patients with primary generalized epilepsy (37%) (26). In contrast, we found no significant difference in baseline depression and anxiety scores, or in the incidence of moderate-to-severe symptoms, in people with focal and generalized epilepsy.

People with epilepsy suffer higher rates of mood disorders than the general population without seizures (25, 27). The biological basis of this comorbidity remains unclear. Do the same changes in brain function that cause the epilepsy predispose patients to psychiatric disorders or, rather, do recurring seizures promote the genesis of psychiatric disorders, either through organic or psychological means? Patients with other chronic diseases have elevated rates of psychiatric disorders, but not as high as in persons with epilepsy (28). Perini et al. (29) reported a frequency of psychiatric diagnoses in patients with temporal lobe epilepsy of 80%, compared to 22% in patients with juvenile myoclonic epilepsy. In addition, impairment of mood can occur before the diagnosis of a seizure disorder (2). A prior diagnosis of depression is associated with a doubling of risk for diagnosis with epilepsy, a poorer prognosis, and a lower chance of remission after temporal lobectomy (4, 30). These data suggest that comorbid mood disorders do not always result solely as a consequence of the initial onset of epilepsy, the occurrence of treatment-resistant epilepsy, or impaired quality of life. These findings suggest that partially overlapping pathologies of brain structure or function, above and beyond psychosocial stressors [e.g., (30)], account for the comorbidity of epilepsy and mood disorders.

### Changes in Mood During the Peri-Ictal Period

In a survey of 100 persons with pharmacotherapy-resistant epilepsy, Kanner et al. (10) reported episodes of postictal depressive events in 43% of patients, characterized by reports of symptoms like anhedonia, frustration, and suicidal ideation, within 24 h after a seizure. Most of the patients had focal temporal seizures and one third had histories of mood disorder diagnoses. The incidence of the symptoms ranged from 13 to 36%, which was higher among the group with prior mood disorder. Symptoms of anxiety in the postictal period were reported by nearly half of epilepsy patients.

We observed that on average, seizures produce a significant *improvement* in mood scores within a 24-h period. Improved depression and anxiety scores were noted following both epileptic and non-epileptic seizures. Among patients with epileptic seizures, only patients with focal onset seizures displayed mood improvements in the peri-ictal period. The improvement in mood after seizures appeared transient and depression and anxiety scores trended back toward the baseline when assessed 2 weeks after the seizure, although the results were not statistically significant. There are numerous differences between our study and that by Kanner et al., including recruitment and inclusion criteria, setting, and exact timing and approach to symptom measurement, but taken together these findings suggest a subset of patients may feel worse especially in those with a clinical history of a mood disorder, whereas on average those with a relatively high quantitative symptom burden will note an improvement.

Our study was not designed to test the effect of specific past diagnosis, but the effect of seizure on current mood symptoms, and therefore future well-planned systematic analyses are needed to better understand the many complex factors that influence these measures. Specifically, larger studies are needed to fully account for duration and severity of both neurological and psychiatric illnesses and the interaction with duration and adequacy of their respective treatments, and the severity of post-ictal neurovegetative symptoms that may influence subjective report.

These consequences of focal epileptic seizures are superficially consistent with the effects of ECT in treating depression. It is usually assumed that the antidepressant effect of ECT results from the induction of seizure activity. A single ECT-induced seizure is insufficient to produce a persistent improvement in mood. Generally, between 5 and 15 ECT treatments are needed for the initial course, and 10–20 maintenance treatments are needed per year to produce a lasting improvement in depression symptoms (31). EEGs are not used to map seizure occurrence in routine ECT. Induction of what are presumed to be generalized seizures is the routine practice in ECT (13, 32, 33) but there is some evidence that focal frontal seizures are equally effective in treating depression (34). Focal temporal seizures are usually avoided, at least initially, because of concerns with interference with temporal lobe memory processes. There is increased evidence from preclinical (35) and human studies (36) that impairments in functional connectivity in reward circuits underlies the genesis of depression. Preclinical and human studies indicate that ECT-like brain stimulation restores circuit function by promoting activity-dependent synaptic plasticity (37, 38). One possible explanation for the transient improvement of mood during the peri-ictal period in ES patients is that similar activity-dependent changes in circuit function occur as a result of seizure activity. It is also possible that the improvement in mood is unrelated to changes in neuronal activity and instead results from psychological factors related to the occurrence of the seizure, such as transient relief from fear of when a seizure will next occur or hope that having a seizure in the EMU will hasten the diagnosis of their epileptic condition.

### Mood and Non-epileptic Seizures

Epileptic seizures result from abnormal, excessive, synchronous neuronal activity. Psychogenic non-epileptic seizures lack electrographic evidence of abnormal brain activity, although the behavioral manifestations of PNES can resemble epileptic seizures. NES patients represented half of the patients in the study despite an incidence of roughly 1 in 10,000 in the general population (17), consistent with previous studies (39). We found that the incidence of moderate-to-severe depression in patients with non-epileptic seizures was comparable to that is people with epileptic seizures (20–30%), but the incidence of moderate-to-severe anxiety was somewhat higher in NES patients than ES patients (43% vs. 30%), as reported previously (15). These results are broadly consistent with previous epidemiological findings (14, 17).

We originally hypothesized that if the altered electrical activity underlying seizures is responsible for the improvement in mood, then only depressed epileptic patients would experience a peri-ictal improvement in mood. Contrary to our original hypothesis, we found, however, that patients with PNES also experienced a reduction in both depression and anxiety scores of 17–34%. PNES remains a poorly understood functional symptom and is currently considered by many to be an unconscious maladaptive psychological coping mechanism. That is, PNES represents a somatization, conversion, or dissociative psychological response that permits unconscious outlet of emotional distress through involuntary physical means. Functional imaging studies (40) reveal an aberrant resting functional connectivity pattern, wherein emotional regulatory centers including the insula and amygdala appropriate greater control over executive function influence on motor centers (40), similar to that observed in dissociative states in trauma-related disorders. We suggest that the transient improvement in mood in response to PNES may result from disproportionate sensitivity and response to the enhancement in attention and care giving in the aftermath of the seizure, thereby providing an outlet for these suppressed psychological stressors similar to other conversion symptoms. There is evidence that events of PNES may decrease after presentation of the diagnosis (41) and this may be a factor contributing to the improvement in mood and anxiety in these patients, as diagnosis was discussed throughout their stay. Indeed, the clinical purpose of a typical EMU stay is to clarify pathophysiology and diagnosis, and thus the act of participating in the admission may operate through a similar neuropsychological mechanism, however further study focusing on PNES is required. These processes may also contribute to the improvement in mood scores in ES patients.

In conclusion, this study demonstrated that post-ictal mood improvements occur in the 24 h following a seizure event but do not persist 2 weeks after the seizure. Furthermore, these improvements differ by seizure type and localization. These findings may help to improve screening for patients with both epilepsy and mood disorders by suggesting that screening should be performed prior to a seizure in the EMU and identifying seizure types more strongly correlated with mood disorders. In addition, this study may help to develop more targeted treatment strategies in this patient population, such as identifying novel target sites and determining the optimal electrical activity for ECT in treating depression.

### Study Limitations

The study has several limitations. First, the patients enrolled in this study are not necessarily representative of the general population of people with epilepsy because they were all evaluated and treated in a tertiary care epilepsy center. Second, the number of patients enrolled who had seizures and completed questionnaires was relatively small (33 patients had epileptic seizures and 31 had non-epileptic seizures). This is problematic given the variance in patients’ baseline mood scores. Our most limited sample was of ES patients with generalized seizure onsets (eight patients). This sample is likely low because this group includes patients with primary generalized epilepsy who may be less likely to need evaluation in an epilepsy monitoring unit for diagnosis. Third, there was a high rate of attrition with patients across the time period we wished to study. Most enrolled patients did not fill out the questionnaires for all the time points within the first 24 h after a seizure event. This may have been due in part to the fact that study evaluation times may have been in the middle of the night when patients did not want to awaken to complete questionnaires. There were challenges in evaluation at the 2-week timepoint as almost all patients were discharged from the hospital prior to this time and may not have returned for follow up. The MADRS questionnaire was particularly challenging to administer. The effect of changing seizure medications may be of importance in the assessment of mood in the EMU. Upon admission to the EMU, antiseizure medications were often changed. Many of these medications may be mood stabilizers or may worsen mood, and the effects of changing medications during the EMU were not measured directly. Comparison of those that did and did not discontinue medications revealed no significant differences, however. Because this was a cross-sectional study in which observations were performed during the course of standard clinical care, it was not possible or ethical to randomize individuals to groups having their meds tapered or not tapered because it would interfere with their clinical care. It is also possible that the admission for testing itself may have an effect. While length of stay in the EMU may potentially impact patient mood, our analysis suggests that this is not a driver of the main effect. The artificial environment itself could affect mood and anxiety and the length of the stay as well as intermittent discussions with a physician about diagnosis could have an impact on these measures. As patients had attention focused on the testing, this may have affected their self-assessment of mood and anxiety during the testing. Some patients may have the expectation that having a seizure will benefit their treatment and the occurrence of such seizures may therefore engender positive psychological outcomes.

We conclude that single seizures, both focal and generalized, result in rapid, transient improvements in mood and anxiety levels in patients with a relatively high quantitative symptom burden and that both epileptic and non-epileptic seizures exert this effect.

## Data Availability Statement

The raw data supporting the conclusions of this article will be made available by the authors, without undue reservation.

## Ethics Statement

The studies involving human participants were reviewed and approved by the University of Maryland Institutional Review Board. The patients/participants provided their written informed consent to participate in this study.

## Author Contributions

JH, ST, and MK designed the study. AP, KG, KT, and MC collected and analyzed the data. JH, AP, MK, and ST wrote and edited the manuscript. All authors contributed to the article and approved the submitted version.

## Conflict of Interest

The authors declare that the research was conducted in the absence of any commercial or financial relationships that could be construed as a potential conflict of interest.

## Publisher’s Note

All claims expressed in this article are solely those of the authors and do not necessarily represent those of their affiliated organizations, or those of the publisher, the editors and the reviewers. Any product that may be evaluated in this article, or claim that may be made by its manufacturer, is not guaranteed or endorsed by the publisher.
